# Supplementary material for: The Acculturation Toolkit: An Orientation for Pediatric International Medical Graduates Transitioning to the United States Medical System
Source: MedEdPORTAL. 2020 Jul 16;16:10922. doi: 10.15766/mep_2374-8265.10922 (PMC7373352; doi:10.15766/mep_2374-8265.10922)
Supplement: Supplementary file 1 — AT Facilitator Overview.docxAT Preworkshop Reflection Questions.docxAT Workshop 1.pptAT Workshop 1 Evaluation.docxAT Workshop 2.pptAT Workshop 2 Role-Play.docxAT Workshop 2 Evaluation.docxAT Workshop 3.pptAT Workshop 3 Role-Play.docxAT Workshop 3 Evaluation.docxAT Workshop 4.pptAT Workshop 4 Role-Play.docxAT Workshop 4 Evaluation.docxAT 1-Year Follow-up Survey.docx [file mep_2374-8265.10922-s001.zip › A. AT Facilitator Overview.docx]

**THE ACCULTURATION TOOLKIT: AN ORIENTATION FOR PEDIATRIC INTERNATIONAL MEDICAL GRADUATES TRANSITIONING TO THE UNITED STATES MEDICAL SYSTEM**

**Facilitator Overview for Implementation**

**Individual Workshop Objectives**

- Workshop 1: The Overview
  1. Explore the differences between doctor-centered and patient-centered models of care
  2. Examine the historical influences on health care dynamics and the doctor-patient relationship in the US
- Workshop 2: The Essentials of Physician-Patient Communication
  1. Understand the importance of effective communication in the doctor-patient relationship
- Workshop 3: The Importance of the Psychosocial History
  1. Use the rapport established from effective doctor-patient communication to gain insight into the medical and social needs of patients
  2. Examine the impact that psychosocial history has on the delivery of health care
  3. Formulate a psychosocial history that will provide insight into the medical needs of a patient
- Workshop 4: Health Literacy
  1. Define health literacy
  2. Apply concept of health literacy to serve our patients better
  3. Design a way to utilize health literacy techniques in different communication scenarios

**Workshop Tools**

- Markers
- Easel pads for group sharing notes
- Easel pads with notes from prior workshops to use in later workshops
- Role Plays for Workshops 2, 3, and 4

**Time Needed for Implementation**

- Workshop 1, “The Overview”: 50-60 minutes
- Workshop 2, “The Essentials of Physician-Patient Communication”: 60-70 minutes
- Workshop 3, “The Importance of the Psychosocial History”: 50-60 minutes
- Workshop 4, “Health Literacy”: 50-60 minutes

**Suggestions for Instructors**

- Facilitator: Preferred facilitators are those with experience in medical education and small group facilitation. Given the support provided in the facilitator’s guide and the instructor’s notes, facilitators do not need to have experience working with residents who are IMGs, but should be sensitive to the challenges of IMGs.
- Scheduling:
  - small groups (no more than 10)
  - multiples of 3 for role play scenarios
  - reserve space
- Send out pre-work reflection questions for participants to consider before the workshop
- Ask participants to submit clinical scenarios that may be incorporated into the workshop discussion (elicit suggestions within the week prior to the workshop)
- Workshops may be conducted in one or multiple settings
- Workshops may work best if delivered in sequence, but also can be delivered asynchronously
- Each presentation includes information in the speaker’s notes section to assist instructors with facilitation
- At the conclusion of each workshop, instructor may want to save the Easel Notes for reference during subsequent workshops
- Instructor should print Role Plays for Workshops 2, 3, and 4. Should have one copy of each Role Play document for every 3 participants
- For Role Plays, several groups can conduct the same scenario if needed
- Test computer/projector set up in room
- Provide food during to the workshops or advise participants to bring food and beverages (depending on time of workshops)

**Suggested Background Reading for Facilitator**

- Osta AD, Barnes MM, Pessagno R, Schwartz A, Hirshfield L. Acculturation needs of pediatric international medical graduates: a qualitative study. *Teach Learn Medicine.* 2017;29(2):143-152.
- Dorgan KA, Lang F, Floyd M, Kemp E. International medical graduate-patient communication: a qualitative analysis of perceived barriers. *Acad Med*. 2009;84(11):1567-75.
- Whelan, G (2006). Commentary: coming to american: the integration of international medical graduates into the american medical culture. *Acad Med.* 2006;81(2):176-178.
- Rao A, Freed C, Trimm RF. International and American medical graduates in a U.S. pediatric residency program: A qualitative study about challenges during post-graduate-year 1. *Med Teach*. 2013;35(10): 815-819.
- Pilotto LS, Duncan GF, Anderson-Wurf J. Issues for clinicians training international medical graduates: a systematic review. *Med J Aust*. 2007;187(4):225-8.
