# Supplementary material for: The Acculturation Toolkit: An Orientation for Pediatric International Medical Graduates Transitioning to the United States Medical System
Source: MedEdPORTAL. 2020 Jul 16;16:10922. doi: 10.15766/mep_2374-8265.10922 (PMC7373352; doi:10.15766/mep_2374-8265.10922)
Supplement: Supplementary file 1 — AT Facilitator Overview.docxAT Preworkshop Reflection Questions.docxAT Workshop 1.pptAT Workshop 1 Evaluation.docxAT Workshop 2.pptAT Workshop 2 Role-Play.docxAT Workshop 2 Evaluation.docxAT Workshop 3.pptAT Workshop 3 Role-Play.docxAT Workshop 3 Evaluation.docxAT Workshop 4.pptAT Workshop 4 Role-Play.docxAT Workshop 4 Evaluation.docxAT 1-Year Follow-up Survey.docx [file mep_2374-8265.10922-s001.zip › B. AT Preworkshop Reflection Questions.docx]

Preworkshop participant reflection questions

| Workshop 1: The Overview | - Where did you train, where have you lived? - How does your background (country, education, religion, prior experience) affect your “lens” of patient care? - Have you had any formal training in patient-doctor communication? - Prior to starting residency, were you worried that you would have challenges communicating with patients? - Did you have any prior experience working with diverse populations as represented by the populations in the US prior to starting residency? |
| --- | --- |
| Workshop 2: The Essentials of  Physician-Patient Communication | - How have you used a patient-centered approach to navigate communication challenges with your patients since the last session? - Has the “patient-centered approach” helped you better handle communication challenges with any of your patients? |
| Workshop 3: Importance of Psychosocial History | - How has residency been going so far?   - Any challenges during interactions with patients?   - Times you have applied elements of the “patient centered” approach? - Updates since last workshop   - Have any of these scenarios from the last session impacted your practice approach since the last workshop?   - Areas of mastery with adjusting to the medical system in the United States?   - Continued challenges with adjusting to medical practice in the United States? |
| Workshop 4: Health Literacy | - Have you seen the themes presented in the last workshop during any of your recent patient care interactions?   - - Managing a “non-adherent parent”     - Understanding contributors to “missed appointments”     - A family who is experiencing “food insecurity” despite obesity |
