# Supplementary material for: The Acculturation Toolkit: An Orientation for Pediatric International Medical Graduates Transitioning to the United States Medical System
Source: MedEdPORTAL. 2020 Jul 16;16:10922. doi: 10.15766/mep_2374-8265.10922 (PMC7373352; doi:10.15766/mep_2374-8265.10922)
Supplement: Supplementary file 1 — AT Facilitator Overview.docxAT Preworkshop Reflection Questions.docxAT Workshop 1.pptAT Workshop 1 Evaluation.docxAT Workshop 2.pptAT Workshop 2 Role-Play.docxAT Workshop 2 Evaluation.docxAT Workshop 3.pptAT Workshop 3 Role-Play.docxAT Workshop 3 Evaluation.docxAT Workshop 4.pptAT Workshop 4 Role-Play.docxAT Workshop 4 Evaluation.docxAT 1-Year Follow-up Survey.docx [file mep_2374-8265.10922-s001.zip › F. AT Workshop 2 Role-play.docx]

**WORKSHOP 2 ROLE PLAYS**

**Scenario #1: Refusal to Vaccine**

**Doctor**

- Mrs. Binder comes to you with her 12 month old son for a well-child visit. You review the records and see that the vaccines have been delayed. You decide to talk to Mrs. Binder about the importance of vaccinations. As you attempt to discuss the 12 month vaccines she states…

“I’m not letting you inject all of these toxins in my child’s body…I agreed to some of these shots, but I don’t want to give my child autism”

- How would you respond to Mrs. Binder?
- What steps would you take to de-escalate the situation?

**Scenario #1: Refusal to Vaccine**

**Patient**

- You are coming in for your 12 month old son’s well child visit.
- When the doctor mentions vaccines you say: “I’m not letting you inject all of these toxins in my child’s body…I agreed to some of these shots I don’t want to give my child autism”

**DO NOT OFFER THIS UNLESS SPECIFICALLY ASKED:**

- Your sister has a son with severe autism and your family is convinced that the 1 year shots caused it

**Scenario # 1: Refusal to Vaccine**

**Observer**:

Describe the doctor’s communication style and approach to the parent’s concerns about autism.

How well did the doctor elicit the parent’s concerns about autism?

How well did the doctor validate the parent’s concerns about autism? Describe how the doctor validated the parent’s concerns.

How did the parent establish a trusting relationship with the parent?

Things done well:

Areas for improvement:

**Scenario #2: Parent Waiting in Clinic**

**Doctor**

- You are about to see your last patient, and you have not had a chance to slow down all morning. The nurse tells you that your patient’s father is upset because he has been waiting over 90 minutes to see you. You finally go in to see Mr. Potter and his 2 year old son. As you walk in, Mr. Potter says, …

“This is why I don’t like coming to this clinic. You don’t respect my time. I took off work this morning to be here, and because I have had to wait so long, now I will be late, and my boss will dock my pay. I am so frustrated!”

**Scenario # 2: Parent Waiting in Clinic**

**Parent**

- You are the father of a 2 year old boy and you have been waiting for 90 minutes to see your child’s doctor. As your doctor walks in you say…

“This is why I don’t like coming to this clinic. You don’t respect my time. I took off work this morning to be here, and because I have had to wait so long, now I will be late, and my boss will dock my pay. I am so frustrated!”

- You are very frustrated because you wait a long time every time you come to the clinic and you were told last time you would be seen first this time.
- It is your son’s nap time so he is extra cranky and has been crying off and on for the 90 minutes.
- You just started a new job and have been told if you are late you could be fired.

**Scenario # 2: Parent Waiting in Clinic**

**Observer**:

How well did the doctor validate the parent’s feelings about the wait? Describe how the doctor validated the parent’s feelings.

How did the doctor respond to the parent’s frustration? Did they respond defensively? Did they apologize?

Things done well:

Areas for improvement:
