# Supplementary material for: The Acculturation Toolkit: An Orientation for Pediatric International Medical Graduates Transitioning to the United States Medical System
Source: MedEdPORTAL. 2020 Jul 16;16:10922. doi: 10.15766/mep_2374-8265.10922 (PMC7373352; doi:10.15766/mep_2374-8265.10922)
Supplement: Supplementary file 1 — AT Facilitator Overview.docxAT Preworkshop Reflection Questions.docxAT Workshop 1.pptAT Workshop 1 Evaluation.docxAT Workshop 2.pptAT Workshop 2 Role-Play.docxAT Workshop 2 Evaluation.docxAT Workshop 3.pptAT Workshop 3 Role-Play.docxAT Workshop 3 Evaluation.docxAT Workshop 4.pptAT Workshop 4 Role-Play.docxAT Workshop 4 Evaluation.docxAT 1-Year Follow-up Survey.docx [file mep_2374-8265.10922-s001.zip › I. AT Workshop 3 Role-play.docx]

**WORKSHOP 3 ROLE PLAYS**

**Scenario # 1: Poorly Controlled Asthma**

**Doctor**:

- Your next clinic patient is Ashley, a 4-year-old coming for follow up after her 3^rd^ asthma hospitalization in 3 months.
- In the notes from the ED there is documentation that Ashley’s mother continues to be non-adherent with the controller medication regimen prescribed.
- You are disappointed with this non-adherence. How do you begin your conversation with Ashley’s mother?

**Scenario # 1: Poorly Controlled Asthma**

**Parent**:

- Ms. Johnson is a single mother who cleans houses.
- She has no one to watch Ashley.
- One of her weekly clients smokes but she has no choice but to bring Ashley to work with her.
- At the job, Ashley is not only exposed to second hand smoke, but the chemical odors from cleaning supplies.
- The insurance company stopped covering the medication.
- She has not had a chance to call you to get authorization from the insurance company so she can pick up Ashley’s medicines.

**(DO NOT OFFER ANY OF THIS INFORMATION UNLESS ASKED ABOUT IT)**

**Scenario # 1: Poorly Controlled Asthma**

**Observer**:

What language did the doctor use in their initial approach to Ashley’s mother?

How did Ashley’s mother respond?

What made this communication style effective? What made it less effective?

Things done well:

Areas for improvement:

**Scenario #2: Multiple Missed Appointments**

**Doctor**:

- Ms. Tipper brings in her 15 month old to get his WIC form completed on a walk-in visit.
- You review the records and realized
  - the infant’s last visit was at 6 months and the 9 month and 12 month visits were missed
  - this child has only been brought in on walk in appointments while the scheduled well child visits are missed
- You are concerned. You walk into the room and introduce yourself. Ms. Tipper greets you by asking, …
  - “Will you fill out the WIC form or not and I know you are going to nag me about the visits I missed.”
- How do you begin your conversation with Ms. Tipper?

**Scenario #2: Multiple Missed Appointments**

**Parent**:

- Ms. Tipper has been in and out of different domestic violence shelters over the last 12 months.
- She has 2 other children (one with autism and 1 with ADHD).
- She is now living an hour by bus away from the clinic.
- She has missed appointments because of various appearances in court related to the domestic violence and school for her son.
- She has no friends or family in the area to help with child care.
- Her 7 year old son just got kicked out of school for bad behavior.

**(DO NOT OFFER ANY OF THIS INFORMATION UNLESS ASKED ABOUT IT)**

**Scenario #2: Multiple Missed Appointments**

**Observer**:

What language did the doctor use in their initial approach to Ms. Tipper?

How did Ms. Tipper respond?

What made this communication style effective? What made it less effective?

Things done well:

Areas for improvement

**Scenario # 3: The Obese Adolescent**

**Doctor**:

- Kevin, a 13-year-old boy is in your office for a weight check with both of his parents. His body mass index is 35, and he has gained 4 pounds since his last visit.
- You have advised dietary changes and exercise at the last visit. You report his weight to the family and ask how they are doing with implementing the recommendations.
- The child tells you that he has been doing his best, but reports being limited by the amount of junk food “tempting him” in the house.
- His father, Mr. Torro shakes his head and states, *“*You doctors think you know everything. What do you know about putting a healthy meal my family’s the table? I make sure my children don’t go hungry by putting dinner on the table every night.”
- How do you begin your conversation with Mr. Torro?

**Scenario # 3: The Obese Adolescent**

**Parent**:

- Mr. Torro works in a fast food restaurant. He struggles with money at the end of the month, and didn’t understand the paperwork to renew his food stamps so lost this benefit.
- He knows the food he provides his family is not the healthiest, but it’s hard to find places for affordable and healthy food in his neighborhood.
- Mr. Torro’s mother watches his kids while he’s at work and is his only childcare option.
- Despite asking his mother to stop feeding the children sugary snacks and high calorie drinks she rewards them with “treats.” She tells him that skinny kids look sick. He is afraid of offending his mother if he speaks up.

**(DO NOT OFFER ANY OF THIS INFORMATION UNLESS ASKED ABOUT IT)**

**Scenario # 3: The Obese Adolescent**

**Observer**:

What language did the doctor use in their initial approach to Mr. Torro?

How did Mr. Torro respond?

What made this communication style effective? What made it less effective?

Things done well:

Areas for improvement:
