# Supplementary material for: The Acculturation Toolkit: An Orientation for Pediatric International Medical Graduates Transitioning to the United States Medical System
Source: MedEdPORTAL. 2020 Jul 16;16:10922. doi: 10.15766/mep_2374-8265.10922 (PMC7373352; doi:10.15766/mep_2374-8265.10922)
Supplement: Supplementary file 1 — AT Facilitator Overview.docxAT Preworkshop Reflection Questions.docxAT Workshop 1.pptAT Workshop 1 Evaluation.docxAT Workshop 2.pptAT Workshop 2 Role-Play.docxAT Workshop 2 Evaluation.docxAT Workshop 3.pptAT Workshop 3 Role-Play.docxAT Workshop 3 Evaluation.docxAT Workshop 4.pptAT Workshop 4 Role-Play.docxAT Workshop 4 Evaluation.docxAT 1-Year Follow-up Survey.docx [file mep_2374-8265.10922-s001.zip › L. AT Workshop 4 Role-play.docx]

**WORKSHOP 4: HEALTH LITERACY**

**ROLE PLAYS**

**Scenario 1: Otitis Media**

**Doctor**:

- You are prescribing amoxicillin/clavulanate to a 5 year old girl with persistent otitis media. You need to give the patient’s mother medication instructions.
- The mother needs to know:
  - Frequency of dosing (1 teaspoon TID)
  - Importance of completing the antibiotic course (10 days)
  - Possible side effects (diarrhea and/or vomiting in 10-20%)
- What do you tell the patient’s mother?

**Scenario 1: Otitis Media**

**Parent**:

You are the parent of a 5 year old girl with persistent otitis media. You never finished high school and don’t read well.

**Scenario 1: Otitis Media**

**Observer**:

How well did the patient’s mother understand the doctor’s instructions?

What strategies did the doctor use to communicate clearly to the patient’s mother?

How effectively did the doctor assess the patient’s mother’s understanding of the instructions?

Things done well:

Areas for improvement:

**Scenario 2: Eczema**

**Doctor**

- You are in clinic seeing a 9 month old with a terrible eczema flare with impetigo. You need to explain the following management to her mother:
  - Cephalexin PO TID
  - Hydrocortisone 2.5% twice daily for inflamed areas on the body
  - Hydrocortisone 1% to the inflamed areas on the face.
  - Mupirocin to the areas of super infection twice a day
  - Diphenhydramine at night for itching
- What do you tell the patient’s mother?

**Scenario 2: Eczema**

**Parent**

You are the mother of a 9 month old with eczema who is having a terrible flare. You have so far just been using Shea Butter on her skin but it’s getting worse.

**Scenario 2: Eczema**

**Observer**:

How well did the patient’s mother understand the doctor’s instructions?

What strategies did the doctor use to communicate clearly to the patient’s mother?

How effectively did the doctor assess the patient’s mother’s understanding of the instructions?

Things done well:

Areas for improvement:

**Scenario 3: Newborn Rule out Sepsis**

Doctor

- You are in the ER seeing a 14 day old baby with a fever.
- You need to explain a full rule out sepsis (blood, urine and lumbar puncture) to the father.
- What do you tell the patient’s father?

**Scenario 3: Newborn Rule out Sepsis**

**Parent**

You are the father of a 14 day old who just brought your baby to the ER for fever.

**Scenario 3: Newborn Rule out Sepsis**

**Observer**:

How well did the patient’s father understand the plan of care for his child?

What strategies did the doctor use to communicate clearly to the patient’s father?

How effectively did the doctor assess the patient’s father’s understanding of the plan of care?

Things done well:

Areas for improvement:
