# Supplementary material for: The Acculturation Toolkit: An Orientation for Pediatric International Medical Graduates Transitioning to the United States Medical System
Source: MedEdPORTAL. 2020 Jul 16;16:10922. doi: 10.15766/mep_2374-8265.10922 (PMC7373352; doi:10.15766/mep_2374-8265.10922)
Supplement: Supplementary file 1 — AT Facilitator Overview.docxAT Preworkshop Reflection Questions.docxAT Workshop 1.pptAT Workshop 1 Evaluation.docxAT Workshop 2.pptAT Workshop 2 Role-Play.docxAT Workshop 2 Evaluation.docxAT Workshop 3.pptAT Workshop 3 Role-Play.docxAT Workshop 3 Evaluation.docxAT Workshop 4.pptAT Workshop 4 Role-Play.docxAT Workshop 4 Evaluation.docxAT 1-Year Follow-up Survey.docx [file mep_2374-8265.10922-s001.zip › M. AT Workshop 4 Evaluation.docx]

**WORKSHOP 4: EVALUATION AND FEEDBACK**

How helpful do you think the following parts of the workshop will be for your future interactions with patient relationships?

|  | Extremely helpful | Helpful | Not sure | Not Helpful | Extremely not helpful |
| --- | --- | --- | --- | --- | --- |
| Reflections from last workshop |  |  |  |  |  |
| Defining Health Literacy |  |  |  |  |  |
| Techniques to improve patient understanding |  |  |  |  |  |
| Defining Teach Back |  |  |  |  |  |
| Role Play: 5 year old with otitis media |  |  |  |  |  |
| Role Play: 9 month old with eczema |  |  |  |  |  |
| Role Play: 14 day old baby with fever |  |  |  |  |  |

THREE things I did not know before this workshop:

1.

2.

3.

THREE practical things I intend to apply my patient care:

1.

2.

3.

THREE things we should do differently for the next workshop:

1.

2.

3.

THREE things I want to learn about in the next workshop:

1.

2.

3.

**I thought this workshop was (circle one):** Too long Too short Just right

**Throughout the workshop, residents had (circle one):**

Too much expectation to talk Not enough opportunity to talk Just right

Rate your enjoyment of the workshop: 1 = WORST EVER, 10 = BEST EVER (circle one)

1 2 3 4 5 6 7 8 9 10

Favorite part:

Least favorite part:

Any other comments?
