# Supplementary material for: The Acculturation Toolkit: An Orientation for Pediatric International Medical Graduates Transitioning to the United States Medical System
Source: MedEdPORTAL. 2020 Jul 16;16:10922. doi: 10.15766/mep_2374-8265.10922 (PMC7373352; doi:10.15766/mep_2374-8265.10922)
Supplement: Supplementary file 1 — AT Facilitator Overview.docxAT Preworkshop Reflection Questions.docxAT Workshop 1.pptAT Workshop 1 Evaluation.docxAT Workshop 2.pptAT Workshop 2 Role-Play.docxAT Workshop 2 Evaluation.docxAT Workshop 3.pptAT Workshop 3 Role-Play.docxAT Workshop 3 Evaluation.docxAT Workshop 4.pptAT Workshop 4 Role-Play.docxAT Workshop 4 Evaluation.docxAT 1-Year Follow-up Survey.docx [file mep_2374-8265.10922-s001.zip › N. AT 1-Year Follow-up Survey.docx]

1. What are three concepts you can recall from the workshops that you think can guide your practice.
2. How (if at all) have the worships impacted your interaction with challenging patients?
3. Please identify and describe at least 1 instance when you drew on something you learned from the workshop.
4. How have the workshops impacted you understanding of Patient Centered Care?
5. How influential were the workshops in improving your understanding of U.S Medical culture? Likert Scale 1-5: Extremely influential (1) to Not at all influential (5)

1 2 3 4 5

1. How influential were the workshops in increasing your confidence in communicating with patients?

Likert Scale 1-5: Extremely influential (1) to Not at all influential (5)

1 2 3 4 5

1. Did the workshops impact your attitudes toward patient care and patients? In what way?
2. Did the workshops impact your satisfaction with your doctor-patient relationships? In what way?

9. How influential were these workshops in making an easier transition to residency?

Likert Scale 1-5: Extremely influential (1) to Not at all influential (5)

1 2 3 4 5
