# Supplementary material for: The Acculturation Toolkit: An Orientation for Pediatric International Medical Graduates Transitioning to the United States Medical System
Source: MedEdPORTAL. 2020 Jul 16;16:10922. doi: 10.15766/mep_2374-8265.10922 (PMC7373352; doi:10.15766/mep_2374-8265.10922)
Supplement: Supplementary file 1 — AT Facilitator Overview.docxAT Preworkshop Reflection Questions.docxAT Workshop 1.pptAT Workshop 1 Evaluation.docxAT Workshop 2.pptAT Workshop 2 Role-Play.docxAT Workshop 2 Evaluation.docxAT Workshop 3.pptAT Workshop 3 Role-Play.docxAT Workshop 3 Evaluation.docxAT Workshop 4.pptAT Workshop 4 Role-Play.docxAT Workshop 4 Evaluation.docxAT 1-Year Follow-up Survey.docx [file mep_2374-8265.10922-s001.zip › _Educational_Summary_Report_10922.pdf]

MedEdPORTAL® The AAMC Journal of Teaching and Learning Resources

Original Publication Open Access

The Acculturation Toolkit: An Orientation for Pediatric International Medical Graduates Transitioning to the United States Medical System

Cynthia Katz, MD*, Michelle Barnes, MD, Amanda Osta, MD, Ingrid Walker-Descartes, MD, MPH, MBA

*Corresponding author: cynthia.katz@mssm.edu

Abstract

Introduction: International medical graduates (IMGs) consistently contribute to the US physician workforce. In fact, 25% of practicing pediatricians in the US are IMGs, highlighting the needs of IMG trainees. IMGs face unique challenges with acculturation compared to their peers due to unfamiliarity with the US medical system, especially the dynamics around patient-centered care. The literature supports the need for formal acculturation curricula. **Methods:** A cohort of program directors who train pediatric IMGs coupled findings from the literature with local themes from IMG focus groups to identify topics for an acculturation curriculum. Three small-group workshops utilized didactics, discussion, and role-play to cover topics related to patient-centered care, challenging communication with patients, complex psychosocial histories, and health literacy. The pilot was modified based on feedback and to enhance generalizability. The resulting four-module curriculum with presentations and supplemental materials is presented here. **Results:** After a 3-year pilot with 36 PGY 1 trainees, postcurriculum surveys reported 8.1 out of 10 in workshop satisfaction, plus increased knowledge and skills related to patient-centered care and communication with patients. Role-plays were the favorite activity. A 1-year follow-up survey reported the workshops to be influential on satisfaction with patient relationships and easing transition to residency. **Discussion:** A pilot acculturation curriculum addressing needs of pediatric IMG trainees was well received by participants and improved their comfort level in addressing challenging patient-communication scenarios. Pediatric programs that train IMGs can incorporate this curriculum to aid residents' transition to clinical practice in the US.

Keywords

Acculturation, International Medical Graduate, IMG, Cultural Competency, Patient-Centered Care, Doctor-Patient Communication, Flipped Classroom, Focus Groups/Interviews, Problem-Based Learning, Diversity, Inclusion, Health Equity

Educational Objectives

By the end of this four-session module, international medical graduate learners will be able to:

1. Demonstrate an improved understanding of US medical culture.
2. Illustrate a shift from a doctor-centered approach to one more focused on patient-centered care.
3. Apply tools to facilitate communication in challenging patient encounters.
4. Demonstrate an increase in confidence in communicating with patients.

Citation:
Katz C, Barnes M, Osta A, Walker-Descartes I. The acculturation toolkit: an orientation for pediatric international medical graduates transitioning to the united states medical system. *MedEdPORTAL*. 2020;16:10922. https://doi.org/10.15766/mep_2374-8265.10922

Introduction

International medical graduates (IMGs) who receive their medical degree outside the US or Canada constitute more than 25% of practicing pediatricians in the US.[1] IMGs also make up a significant portion of the workforce in graduate medical education in the US. In the 2018 match, residents who were born outside the US and graduated from medical school outside the US (non-US IMGs) constituted 12% of matched applicants into pediatrics residency programs[2] and 14% of matched applicants to pediatric fellowships.[3] Some IMGs enter fellowships prior to completing a residency training program in the US, making the needs of these trainees a pertinent issue for fellowship program directors as well. Upon entering their training programs, IMGs face unique acculturation challenges, especially in physician-patient communication[4,5] and patient-centered medicine.[6-8] Published work on this topic focuses on two themes: (1) understanding IMGs' specific needs and challenges and (2) proposing strategies to support IMGs through the improvement of

Copyright © 2020 Katz et al. This is an open-access publication distributed under the terms of the Creative Commons Attribution license.

communication and language skills.[9] Other than these areas, a broad literature search revealed little guidance outlining a specific acculturation curriculum to introduce and instill the necessary competencies during pediatric training.[10] To date, *MedEdPORTAL* has published two curricula pertaining to IMGs: one addressing internal medicine residents' chart documentation skills,[11] and the other addressing asking difficult questions, with only a partial focus on IMGs.[12] Neither specifically addresses acculturation.

The literature highlights the need for a deliberate acculturation curriculum for IMGs in their transition to the US medical system.[13] The needs that IMG trainees have include lack of familiarity with the US medical system, adjusting to a new culture, and mastering the nuances of communication with specific patient populations.[14-17] Prior to coming to the US, IMG residents have been socialized to the culture of their home country. Upon arrival, they must adjust to a new culture in the US, to the practice of medicine in the US, and, even more specifically, to the culture of pediatrics.[13] The resulting response is the Acculturation Toolkit, a four-module acculturation curriculum that serves to address the unique needs of most IMG trainees matched to pediatric programs across the country.

The Acculturation Toolkit was developed by our team of pediatric program and associate program directors. We are not IMGs ourselves, but each of us has extensive experience training, mentoring, and remediating pediatric IMGs. We utilized themes in the literature, as well as personal experiences training IMGs, to home in on vital topics specific to IMGs. The purpose of this resource is to address a unique curricular gap for IMG residents, with the long-term objective of improved patient outcomes. The topics identified in this curriculum are instrumental in addressing service gaps and health disparities for many underserved patient populations.[18] We, the authors of this curriculum, are a representative sample of program directors from training programs across the country, and thus, this curriculum is intended to be generalizable. The needs of IMGs are by no means homogeneous, as the broader acculturation literature demonstrates the variability with which immigrants adapt to new cultures, based on the cultural differences between the countries of origin and the host countries, as well as personal factors.[19]

These materials are intended to allow any educator to implement and facilitate the curriculum. The curriculum can be stand-alone, or portions can be adapted to enhance any existing cultural competency curriculum where improved communication strategies may be useful.

Methods

Development

A pilot of the Acculturation Toolkit was implemented for 3 consecutive years in a community hospital-based residency program serving a multicultural urban underserved patient population. To standardize the delivery of the content, the same faculty member led the workshop for 3 years. The pilot curriculum was presented as three modules, but the current, modified version of the Acculturation Toolkit consists of four modules. In preparation for dissemination of the Acculturation Toolkit, the group of collaborating program directors reviewed the materials from the pilot curriculum's three modules. To add to the generalizability of the curriculum, some topic areas were expanded, and a few new topics were added. The modifications included (1) a deeper dive into the historical context for mistrust of the medical community, (2) a section on conflict resolution and de-escalation techniques, and (3) enhanced role-plays. In order to keep discrete, deliverable, hour-long workshops, the modules were reconfigured into the four-module version described in this publication.

Target Audience

The learners were categorical pediatric interns (PGY 1s) within their first 3 months of residency training in the US. Each class consisted of 12 PGY 1s who had trained in various countries as well two or three non-IMG participants. The decision was made not to exclude non-IMGs from participating alongside their IMG colleagues because of anticipated mutual benefits. First, exposure to the curriculum would help facilitate non-IMGs to develop a better understanding of the unique challenges faced by their IMG colleagues and therefore create a more cohesive and sympathetic learning environment for all trainees. Second, including all PGY 1s would allow for sharing perspectives and support a more productive dialogue.

Ideally, facilitators should have experience in medical education and small-group facilitation. Given the support provided in the facilitator's guide (Appendix A) and the instructor's notes within the presentations, facilitators do not need to have experience working with residents who are IMGs but should be sensitive to the challenges of IMGs. Appendix A includes some suggested background reading for facilitators new to working with IMGs.

Implementation

It is highly recommended that the Acculturation Toolkit be initiated during the first 2 months and completed within the first 6 months of the academic year. In our experience, this early participation served to balance the newness of clinical

Copyright © 2020 Katz et al. This is an open-access publication distributed under the terms of the Creative Commons Attribution license.

exposure with some familiarity with clinical experience on the part of the trainees, yet before they acquired substantial practice experience. Preworkshop practice experience gave the trainees context for the reflection exercises and facilitated a rich discussion using experiences from the attendees.

Twelve PGY 1s, divided into two groups of six, participated in all three workshops. For ease of scheduling, the groups were shuffled between all workshops, and the same six PGY 1s did not participate in all workshops together. Facilitators found that groups of six worked well for candid discussions, but the general recommendation for small-group work is to have no more than 10 trainees in a group in order to enhance cohesive group dynamics for discussion.

Prior to each workshop, the facilitator sent trainees a set of reflection questions (Appendix B) in order to allow trainees to formulate thoughts and enhance discussion flow. Each workshop ran for approximately 60 minutes. The workshops were run in sequential order but could be rearranged if needed. About a week prior to each workshop, facilitators asked participants to submit clinical scenarios that may be incorporated into the workshop discussion. In preparation for each workshop, the facilitators reviewed the speaker notes within each PowerPoint to plan discussion points, made copies of the role-play scenarios (separated into the three roles), reserved the room space, tested the computer/projector setup in the room, and if the workshop occurred over the lunch hour, planned for food provision or advised participants to bring food and beverages. Each workshop used some combination of didactics, self-reflection, pair-share, small-group facilitated discussion, and case-based role-play in order to provide sufficient content while keeping participants engaged throughout.

Workshop 1: The Overview (Appendix C) focused on participants sharing background and experiences, introduced the concept of patient-centered care, and presented the historical context for mistrust in the medical profession by certain groups using a mixture of didactic and small- and large-group discussions. An easel was used throughout the workshop to write down notes, which were used to review the Workshop 1 material during Workshop 2. Following the workshop, learners completed the evaluation form (Appendix D).

Workshop 2: The Essentials of Physician-Patient Communication (Appendix E) focused on challenging patient scenarios, conflict, and de-escalation strategies. Following a large-group discussion, learners broke into groups of three to complete a 15-minute role-play activity (Appendix F). The two role-play scenarios were vaccine refusal and parents frustrated with the wait time in clinic. As each scenario had three roles (doctor, parent, observer), we broke learners into small groups of three and had all groups review the same scenario. The roles were rotated every 5 minutes so that each participant served in all three roles for the scenario, and then, the small groups reported out to the larger group. Prior to leaving the session, all learners completed the Workshop 2 evaluation form (Appendix G).

Workshop 3: The Importance of the Psychosocial History (Appendix H) covered the why and how of psychosocial history using a combination of didactic, large-group, think-pair-share, and small-group role-play modalities. The three role-play scenarios (Appendix I) were about poorly controlled asthma, multiple missed appointments, and an obese adolescent. Each scenario included doctor, parent, and observer roles. Following a debrief of the role-play, trainees completed the Workshop 3 evaluation (Appendix J).

Workshop 4: Health Literacy (Appendix K) defined and discussed health literacy and the teach-back method. Following a discussion-based didactic on the two concepts, participants broke up into small-groups to review the role-play cases. The role-play scenarios (Appendix L) were about a 5-year-old with otitis media, a 9-month-old with eczema, and 14-day-old with fever. Each scenario included doctor, parent, and observer roles. Following a debrief of the role-play, trainees completed the Workshop 4 evaluation (Appendix M).

Assessment and Evaluations

During the last 10 minutes of each workshop, participants were asked to complete evaluations containing both quantitative and qualitative elements to assess utility of content presented and satisfaction with the workshop structure and to elicit feedback to apply to future workshops (Appendices D, G, J, and K). Quantitative data from the workshop evaluation forms were pooled from the 3 years of data and assessed for mean satisfaction ratings, satisfaction with time allotted and opportunity to talk, and ratings for utility toward future patient interactions. Qualitative data were analyzed for themes to inform adaptations to the curriculum from year to year.

All participants were asked to complete the Patient-Practitioner Orientation Scale (PPOS)[20] before and after administration of the workshop series. A validated tool for assessing beliefs about patient-centeredness, the PPOS was used with permission from its author and creator. Literature supports the idea that medical practice outside the US is less focused on patient-centered care and more focused on doctor-centered care. Patient-centered

Copyright © 2020 Katz et al. This is an open-access publication distributed under the terms of the Creative Commons Attribution license.

care is a significant theme threaded through the Acculturation Toolkit. The PPOS was chosen to assess the impact of the workshops on trainees' perception of the roles of giving and receiving medical care by the physician and patient, respectively. PPOS scores were pooled for the 3 years of implementation and analyzed using a Wilcoxon signed rank test to assess change before and after the completion of all three workshops.

Participants were also surveyed (Appendix N) via SurveyMonkey 1 year after the completion of workshop participation to assess sustained impact of the curriculum.

Results

Thirty-six PGY 1 trainees (12 per academic year) participated in the three-workshop version of the curriculum included in our pilot acculturation curriculum. Over the 3 years of implementation, participants represented 14 different countries of medical training, which are listed in the Table. All participants completed the pre- and post-PPOS, as well as four postworkshop evaluations. PPOS scores revealed a statistically significant postcurriculum increase in importance of patient-centered care ($W = 10$, $p = .048$). Mean satisfaction ratings for each of the three workshops on a 10-point Likert scale (1 = *worst ever,* 10 = *best ever*) were 8.4, 7.5, and 8.5, with "Just right opportunity to talk" as the most common sentiment expressed. Ratings for each workshop component were between extremely helpful (1) and helpful (2) on a 5-point Likert scale (1 = *extremely helpful,* 5 = *extremely not helpful*) for future patient interactions. Participants anticipated applying workshop content to challenging communication scenarios with patients, addressing challenges in communication with patients of different cultures, and aiming for patient-centered care. The role-play exercises were reported as the favorite component and described as "useful tools for application to clinical care."

Table. Number of Participants per Cohort by Country of Training

Country	2013-2014	2014-2015	2015-2016
China	1		1
Egypt			1
India	1	2	3
Iraq			1
Jamaica			1
Korea		1	
Latvia		1	
Nepal	2	1	
Nigeria	2	2	
Pakistan	1	1	2
Palestine	1		
Syria	1		
Taiwan		1	1
United States	3	3	2

The 1-year follow-up survey had a 58% completion rate. Residents reported retained knowledge and skills in areas of improved understanding of patient-centered care, patient communication, and skills for encounters with challenging patients. All (100%) respondents assessed the curriculum as extremely (1) to moderately (2) influential on a 5-point Likert scale (1 = *extremely influential,* 5 = *not at all influential*) on improving understanding of US medical culture, increasing confidence in communicating with patients, and making for an easier transition to residency. Qualitative responses from the 1-year follow-up surveys fell into three themes:

- Improved understanding of patient-centered care:
  - "I know it's OK to say, 'I don't know."'
  - "I use teach back."
  - "I now make sure to identify who is present."
  - "I now ask about social conditions more."
  - "Approach every patient with respect."
- Patient communication:
  - "I try to better understand patients and parents."
  - "I try not to use prejudgments."
  - "I try to understand patients' socioeconomic status."
  - "Now I give more time to parents' concerns to make them understand best possible."
- Skills for encounters with challenging patients:
  - "The scenario from the last session was helpful because I had two experiences when patients were not happy with waiting time."
  - "I can now tactfully handle an angry parent."

Discussion

In the development of the Acculturation Toolkit, the collaborating program directors identified a gap in the preparation of their trainees who had received their preresidency training outside the US. The themes presented in this curriculum successfully addressed areas of need for IMGs. The availing curriculum is not only generalizable but flexible, so that any facilitator can adapt the original content to address needs that might be unique to the training site and the populations served. Individual programs differ in the cultural identity of trainees, and therefore, facilitators may need to emphasize and deemphasize different parts of the modules to maximize their effectiveness in mitigating any challenges to communication posed by patient-provider differences.

Thus far, components of this curriculum have been incorporated into a separate workshop at a national conference to enhance concepts related to acculturation for IMGs. Additionally, several

Copyright © 2020 Katz et al. This is an open-access publication distributed under the terms of the Creative Commons Attribution license.

abstracts utilizing various components of the Acculturation Toolkit have been presented at national conferences. In these forums, the Acculturation Toolkit has generated rich discussions about the complex issues that arise while training IMGs, including challenges faced by both residents and faculty, as well as the varying approaches considered to address acculturation issues. We have received numerous requests from educators in program leadership, medical trainees at all levels, and program coordinators to share our curriculum materials. We are pleased to make the Acculturation Toolkit available for programs to use to support the transition of their IMG trainees.

Limitations, Challenges, and Future Directions

The primary limitation of our study is that the data presented here are all from a single site and reflect only the three-workshop pilot version of the curriculum. This was not a multisite study, and therefore, we do not intend to overgeneralize the findings. However, we are from multiple sites and hope to successfully inform how this curriculum can translate into a broad variety of learning environments. After analysis of participant feedback from the original workshops, as well as discussion among ourselves, the Acculturation Toolkit developed into a more generalizable and usable set of materials for a wider range of programs. The curriculum is now better suited to be implemented in a variety of programs that train a broad demographic of IMGs, as well as those program sites that serve a more diverse patient population. We plan to continue to implement the Acculturation Toolkit with current IMG trainees at our program sites with the intention of studying the impact of the curriculum on residents' interpersonal and communication skills with their patients.

We recognize several other limitations relating to the curricular evaluations. First, although the evaluations were completely anonymous, there were only six participants in each group, and therefore, participants may not have felt anonymous enough to be completely honest. Additionally, the faculty member facilitating the workshops was one of the associate residency program directors with whom the participants worked closely both clinically and administratively. In our case, this was the faculty member best qualified to facilitate the workshops. However, it is possible that trainees would have felt too vulnerable to give negative evaluations and would have a fear of retaliation from the faculty member. The possibility of a more neutral facilitator should be explored by other programs looking to eliminate the possibility of biased evaluations. Finally, the assessments used to measure satisfaction and impact of the curriculum, including the pre- and post-PPOS, were based on self-reported data. By definition, self-reported data introduce bias. Also, using self-reported data does not assess actual behavioral change but rather reports perceived behavior change. Further study with OSCEs, direct observations, and patient reports would be helpful for investigating curricular impact on actual behavior change as well as impact on patient care.

A limitation of the data is that they were analyzed with IMG and non-IMG residents together, reflecting the way the curriculum was implemented and evaluated. As described above, workshops were facilitated in small groups of six, and evaluations were anonymous. Due to the small overall sample size and the even smaller number of IMG residents in each group, we did not ask the trainees to identify their status as IMG or non-IMG in order to maintain their anonymity. We prioritized anonymity to protect the identities of our participants as well as to facilitate more candid evaluations. Separate data analysis for IMGs and non-IMGs could be something to consider in future iterations of the workshop evaluations, particularly with an increased sample size.

After the pilot of this curriculum and local institutional dissemination of the results, the Acculturation Toolkit was adapted and implemented for the internal medicine residents. Anecdotally, the internal medicine residents also found the workshops engaging and useful, with similar mean satisfaction scores as the pediatric trainees, although no formal evaluation took place. Since we have no formal evaluation, we are unable to accurately extrapolate whether IMG trainees of any other subspecialty would benefit from the subject matter, especially as it pertains to familiarity with US medical culture and patient-centered care and communication. We welcome collaboration with our nonpediatric colleagues to implement the curriculum and help gather data to show benefit for IMG trainees in other specialties.

One challenge for implementation is scheduling the optimal time. Capturing all first-year trainees after the start of busy clinical work may prove challenging. Another option for programs is to implement this four-workshop curriculum during program orientation. This approach may come with its own set of difficulties as the preworkshop clinical experience may not be consistent across IMG participants. If trainees have had observership experiences in the US, they will have some clinical context for discussion. For those trainees who have not had any US clinical experience and therefore lack the clinical context we are trying to establish, it would be more appropriate to implement the workshops later. Additionally, it is not uncommon for IMG residents to have a delay in starting residency, due to a holdup in visa issuance. This delay could cause IMG residents to miss the workshops if they occur in orientation or early in

Copyright © 2020 Katz et al. This is an open-access publication distributed under the terms of the Creative Commons Attribution license.

the academic year. Trainees with visa-based delays could be assigned prework requiring them to view the workshop overview individually, thereby contextualizing the subsequent tasks, and to contemplate some of the slides outlining specific questions and the role-play exercises. After arriving and initiating clinical work, these trainees could then participate in the facilitated workshops. Other technological web-based learning platforms to accommodate distance learning could also be a consideration to address these challenges.

Like ours, many programs across the US have a combination of non-US (or foreign-born) IMGs, US IMGs, and American medical graduates, all with varying degrees of US clinical experience. During the 3 years of implementation of the pilot acculturation curriculum, participants came from each of these three categories of trainees. As discussed above, programs with trainees with diverse training backgrounds should consider having all trainees participate together in the curriculum for mutual benefit. Although certain content will be more familiar to some trainees, many curricular topics may be novel, and the benefits of hearing about experiences and reflecting on perspectives of foreign-born colleagues cannot be overstated. An opportunity for future evaluation could focus on reflections on how the non-IMG learners receive this content.

Conclusion

A rigorous needs assessment utilizing focus groups and a literature review led to the development of the Acculturation Toolkit curriculum and supporting materials. Participant evaluation and feedback from our pilot acculturation curriculum suggest that goals were met, and 1-year follow-up demonstrated sustained impact on self-reported resident behavior. We recommend that pediatric programs that train IMGs incorporate the Acculturation Toolkit to support IMGs when cultural nonalignment may pose a challenge in the early stages of patient engagement.

Appendices

A. AT Facilitator Overview.docx
B. AT Preworkshop Reflection Questions.docx
C. AT Workshop 1.ppt
D. AT Workshop 1 Evaluation.docx
E. AT Workshop 2.ppt
F. AT Workshop 2 Role-Play.docx
G. AT Workshop 2 Evaluation.docx
H. AT Workshop 3.ppt
I. AT Workshop 3 Role-Play.docx
J. AT Workshop 3 Evaluation.docx
K. AT Workshop 4.ppt
L. AT Workshop 4 Role-Play.docx
M. AT Workshop 4 Evaluation.docx
N. AT 1-Year Follow-up Survey.docx

All appendices are peer reviewed as integral parts of the Original Publication.

Cynthia Katz, MD: Assistant Clinical Professor, Department of Pediatrics, Icahn School of Medicine at Mount Sinai; Associate Residency Program Director, Department of Pediatrics, Icahn School of Medicine at Mount Sinai

Michelle Barnes, MD: Associate Professor, Department of Pediatrics, University of Illinois College of Medicine; Associate Residency Program Director, Department of Pediatrics, University of Illinois College of Medicine

Amanda Osta, MD: Associate Professor, Department of Pediatrics, University of Illinois College of Medicine; Residency Program Director, Department of Pediatrics, University of Illinois College of Medicine

Ingrid Walker-Descartes, MD, MPH, MBA: Assistant Professor, Department of Pediatrics, State University of New York Downstate Medical Center College of Medicine; Residency and Fellowship Program Director, Department of Pediatrics, Maimonides Infants and Children's Hospital of Brooklyn

Acknowledgments

We would like to acknowledge the contribution of the Academic Pediatric Association Educational Scholars Program Cohort 5 (2012-2015), which includes Virginia Niebuhr, PhD, Steve Paik, MD, EdM, Kenneth Bromberg, MD, Sarah Rawstron, MBBS, and Johanna Bromberg-Craig, PhD.

Disclosures

None to report.

Funding/Support

None to report.

Prior Presentations

Katz C, Laila S, Trappani L, Rawstron S. A "focus" on IMG communication. Poster presented at: APPD Annual Spring Meeting; April 2-5, 2014; Chicago, IL.

Katz C, Bromberg-Craig J, Niebuhr V. Strangers in strange land: an acculturation curriculum for international medical graduates. Poster presented at: APPD Annual Spring Meeting; March 25-28, 2015; Orlando, FL.

Copyright © 2020 Katz et al. This is an open-access publication distributed under the terms of the Creative Commons Attribution license.

Katz C, Barnes M, Osta A, Paik S, Trimm F. Strangers in a strange land: building an acculturation curriculum for international medical graduates. Workshop presented at: APPD Annual Spring Meeting; March 25-28, 2015; Orlando, FL.

Katz C, Bromberg-Craig J, Niebuhr V. Strangers in strange land: an acculturation curriculum for international medical graduates. Poster presented at: Pediatric Academic Societies Annual Meeting; April 25-28, 2015; San Diego, CA.

Katz C. Strangers in a strange land: a cultural competency curriculum for international medical graduates. Presented at: Underrepresented Minorities in Pediatric GME Special Interest Symposium, APPD Annual Spring Meeting; March 30-April 2, 2016; New Orleans, LA.

Ethical Approval

The Brooklyn Hospital Center Institutional Review Board approved this study.

References

1. About the International Medical Graduates Section (IMGS). American Medical Association website. Accessed June 13, 2018. https://www.ama-assn.org/about-us/about-international-medical-graduates-section-imgs
2. National Resident Matching Program. *Results and Data: 2018 Main Residency Match*. National Resident Matching Program; 2018.
3. National Resident Matching Program. *Results and Data: Specialties Matching Service 2018 Appointment Year*. National Resident Matching Program; 2018.
4. Jain P, Krieger JL. Moving beyond the language barrier: the communication strategies used by international medical graduates in intercultural medical encounters. *Patient Educ Couns*. 2011;84(1):98-104. https://doi.org/10.1016/j.pec.2010.06.022
5. Searight HR, Gafford J. Behavioral science education and the international medical graduate. *Acad Med*. 2006;81(2):164-170.
6. Tromp F, Rademakers JJDJM, Ten Cate TJ. Development of an instrument to assess professional behaviour of foreign medical graduates. *Med Teach*. 2007;29(2-3):150-155. https://doi.org/10.1080/01421590601178014
7. Charles C, Gafni A, Whelan T. Shared decision-making in the medical encounter: what does it mean? (or it takes at least two to tango). *Soc Sci Med*. 1997;44(5):681-692. https://doi.org/10.1016/S0277-9536(96)00221-3
8. Slowther A, Lewando Hundt GA, Purkis J, Taylor R. Experiences of non-UK-qualified doctors working within the UK regulatory framework: a qualitative study. *J R Soc Med*. 2012;105(4):157-165. https://doi.org/10.1258/jrsm.2011.110256
9. Chen PGC, Curry LA, Bernheim SM, Berg D, Gozu A, Nunez-Smith M. Professional challenges of non-U.S.-born international medical graduates and recommendations for support during residency training. *Acad Med*. 2011;86(11): 1383-1388. https://doi.org/10.1097/ACM.0b013e31823035e1
10. Lineberry M, Osta A, Barnes M, Tas V, Atchon K, Schwartz A. Educational interventions for international medical graduates: a review and agenda. *Med Educ*. 2015;49(9):863-879. https://doi.org/10.1111/medu.12766
11. Talwalkar J, Ouellette J. A structured workshop to improve chart documentation among housestaff. *MedEdPORTAL*. 2009;5:5095. https://doi.org/10.15766/mep_2374-8265.5095
12. Hahn-Cover K. The patient encounter: asking difficult questions. *MedEdPORTAL*. 2007;3:540. https://doi.org/10.15766/mep_2374-8265.540
13. Osta AD, Barnes MM, Pessagno R, Schwartz A, Hirshfield LE. Acculturation needs of pediatric international medical graduates: a qualitative study. *Teach Learn Med*. 2017;29(2):143-152. https://doi.org/10.1080/10401334.2016.1251321
14. Vamos M, Watson N. Coming on board: the assessment of overseas trained psychiatrists by the Royal Australian and New Zealand College of Psychiatrists. *Australas Psychiatry*. 2009; 17(1):38-41. https://doi.org/10.1080/10398560802469736
15. Hall P, Keely E, Dojeiji S, Byszewski A, Marks M. Communication skills, cultural challenges and individual support: challenges of international medical graduates in a Canadian healthcare environment. *Med Teach*. 2004;26(2):120-125. https://doi.org/10.1080/01421590310001653982
16. Rao A, Freed CR, Trimm RF. International and American medical graduates in a U.S. pediatric residency program: a qualitative study about challenges during post-graduate year 1. *Med Teach*. 2013;35(10):815-819. https://doi.org/10.3109/0142159X.2013.802297
17. Karnik AM. International medical graduates—training all over again. American College of Physicians website. Published 2000. Accessed June 13, 2018. https://www.acponline.org/about-acp/acp-international/for-international-medical-graduates/training-in-the-us/differences
18. Mick SS, Lee SYD. The safety-net role of international medical graduates. *Health Aff (Millwood)*. 1997;16(4):141-150. https://doi.org/10.1377/hlthaff.16.4.141
19. Berry JW. Immigration, acculturation, and adaptation. *Appl Psychol*. 1997;46(1):5-34. https://doi.org/10.1111/j.1464-0597.1997.tb01087.x
20. Trapp S, Stern M. Critical Synthesis Package: Patient-Practitioner Orientation Scale (PPOS). *MedEdPORTAL*. 2013;9:9501. https://doi.org/10.15766/mep_2374-8265.9501

Received: April 10, 2019
Accepted: December 26, 2019
Published: July 16, 2020

Copyright © 2020 Katz et al. This is an open-access publication distributed under the terms of the Creative Commons Attribution license.
